# Modeling of inter-neuronal coupling medium and its impact on neuronal synchronization

**DOI:** 10.1371/journal.pone.0176986

**Published:** 2017-05-09

**Authors:** Muhammad Iqbal, Muhammad Rehan, Keum-Shik Hong

**Affiliations:** 1 Department of Computer and Information Sciences, Pakistan Institute of Engineering and Applied Sciences (PIEAS), Islamabad, Pakistan; 2 Department of Electrical Engineering, Pakistan Institute of Engineering and Applied Sciences (PIEAS), Islamabad, Pakistan; 3 Department of Cogno-Mechatronics Engineering and School of Mechanical Engineering, Pusan National University, Geumjeong-gu, Busan, Republic of Korea; Lanzhou University of Technology, CHINA

## Abstract

In this paper, modeling of the coupling medium between two neurons, the effects of the model parameters on the synchronization of those neurons, and compensation of coupling strength deficiency in synchronization are studied. Our study exploits the inter-neuronal coupling medium and investigates its intrinsic properties in order to get insight into neuronal-information transmittance and, there from, brain-information processing. A novel electrical model of the coupling medium that represents a well-known RLC circuit attributable to the coupling medium’s intrinsic resistive, inductive, and capacitive properties is derived. Surprisingly, the integration of such properties reveals the existence of a natural three-term control strategy, referred to in the literature as the proportional integral derivative (PID) controller, which can be responsible for synchronization between two neurons. Consequently, brain-information processing can rely on a large number of PID controllers based on the coupling medium properties responsible for the coherent behavior of neurons in a neural network. Herein, the effects of the coupling model (or natural PID controller) parameters are studied and, further, a supervisory mechanism is proposed that follows a learning and adaptation policy based on the particle swarm optimization algorithm for compensation of the coupling strength deficiency.

## Introduction

The neuron is an innate sophisticated structural entity of any nervous system. Contemporary research investigates its chief biophysical features and key mechanisms of operations for effective transmission of neuronal signals between the brain and the muscles. The probe of neuron doctrine has emerged as an important research area in the field of neuroscience that provides insight into brain-information processing and information transmittance among neurons [1–8]. Neural-system malfunctions can contravene many physiological brain functions such as neuro-signal transmittance, thus potentially resulting in various neuronal diseases such as Parkinson’s, Huntington’s, and epilepsy [9–11].

Given the infeasibility of measuring neurological processes, especially neuro-signal transmission, investigation of the underlying mechanisms of transmitting media remains as a contemplated research subject. The coupling medium (that is, extracellular medium) between neurons, through which they communicate, exhibits electrical characteristics from the view that many neuronal activities are electrical in nature. To a certain extent, endeavors have been devoted to the investigation of the electrical behavior of the coupling medium, specifically by exploration of its resistive characteristics [12–17]. Nonetheless, such studies provide a simple model on the coupling strength; but these studies do not incorporate the unknown biological processes in modeling the coupling medium using electrical components, which can result in restrictive models.

Modern-day biological studies have shown that the coupling medium (or extracellular medium) between two neurons has resistive, capacitive, and inductive properties [17–20]. Some theoretical and experimental studies conclude that the extracellular medium possesses the capacitive features. One of the existing theoretical and experimental works includes non-resistive extracellular media instead of the traditional resistive extracellular media and proposes that the scaling of EEG and MEG signals can be reconciled through capacitive characteristics of the extracellular medium [18]. Moreover, various theoretical studies regarding the cable theory, which is one of the most noteworthy contributions in neuroscience, also considered the capacitive properties of the extracellular media other than the resistive media [19]. In which, the authors generalized the cable equations to illustrate the neuronal membranes surrounded by arbitrarily complex and heterogeneous extracellular media. The demonstrated results revealed that the extracellular media can exhibit other complex electrical characteristics like capacitive effect and diffusion, and have drastic impacts on fundamental cable properties. Another research [20] showed that the extracellular medium has the inductive nature as well. Such inductive coupling involves modifying neighboring neurons by the ion currents in the extracellular medium from the conducting neuron in close opposition. These findings motivate us to develop a sophisticated mathematical model of the inter-neuronal coupling medium and to examine the coupling behavior of neurons due to the medium characteristics.

Accurate modeling of the coupling medium is essential to any investigation of neuronal transmission. This is because it plays a critical role in their synchronization [21–24], which is a fundamental phenomenon for information processing and integration in neural systems. Studies dealing with the neuronal modeling along with the relevant extracellular medium properties and synchronization under external electrical stimulation are also essential in effort to discover cures for brain disorders [25–27]. In the recent studies associated with the neuronal modeling [28–30], complex dynamical behavior of electrical activities in the neuronal systems has been explored by considering the effect of Faraday’s law of electromagnetic induction using magnetic fluxes. Consequently, realistic and improved neuronal models are developed that can be utilized for understanding of synchronization process in brain. Another simple model is the FitzHugh-Nagumo (FHN) model under external electrical stimulation, which has been considered owing to its utility in representing the dynamical behavior of neurons and its usefulness in describing and exploring the inter-neuronal coupling medium [31–33]. It is worth noting that studies on accurate modeling of the inter-neuronal medium and neuronal behavior under different medium couplings have not been fully addressed in the literature yet [34–37].

The present study exploits the neuronal coupling medium in order to investigate its diverse electrical behavior for better understanding of information transmittance among neurons and muscles. Modeling of the inter-neuronal coupling medium, the effects of the coupling medium on synchronization between neurons, and learning and adaptation policy based mechanisms for synchronization of FHN neurons are explored. The curiosity in the coupled neuronal system and neural networks is more often than not due to the complicated behavior of the medium: Indeed, the transmittance of neural signals to different regions in the brain is highly sensitive to the medium’s coupling properties. A novel electrical model of the coupling medium that represents a well-known parallel RLC circuit attributable to the medium’s intrinsic resistive, inductive and capacitive properties, among others, is introduced. The coupling medium properties are related to resistors, capacitors, and inductors owing to the motivations from the existing studies and due to physical characteristics investigated through Faraday’s law of electromagnetic induction. Existence of a parallel RLC circuit due to coupling between neurons is proposed on the basis of superposition principle and the hypothesis of linear behavior of the coupling medium for simplicity purpose.

It is worth noting that incorporation of the proposed coupling medium model reveals the existence of a natural three-term controller, which is well known as the proportional integral derivative (PID) controller. Fundamentally, a PID structure consists of proportional, integral, and derivative parameters that can be utilized for control of the transient and the steady-state behaviors of a system to attain a desired response [38]. Upon the viability and existence of natural PID properties, the effects of coupling properties of the medium in terms of the natural PID components on the synchronization of FHN neurons are studied. Beside the study on a natural mechanism, a supervisory mechanism that can compensate for the natural coupling strength deficiency, specifically by employing a learning and adaptation policy in order to tune PID control parameters utilizing a particle swarm optimization (PSO) algorithm, is developed for synchronization assurance. This supervisory mechanism will present a stark contrast to the traditional schemes, see [39–41]. The results of the proposed approach are verified through numerical simulation results.

The remainder of the paper proceeds as follows: Section 2 discusses the main results, which include a new modeling approach to the neuronal coupling medium, the theoretical reasoning for medium properties, the discovery of the existence of a natural PID controller, the effects of the coupling properties of the medium in terms of natural PID components on neuronal synchronization, and the compensation for the natural coupling strength deficiency by utilization of a supervisory mechanism of PID-controlled-synchronization. Section 3 addresses the utility of the employed methods, application of Faraday’s law, superposition principle, the FHN model, synchronization of nonlinear systems, the PID control approach, and PSO algorithm. Section 4, finally, draws conclusions.

## Results and discussion

### Theoretical reasoning for inductive coupling

**Fig 1:**
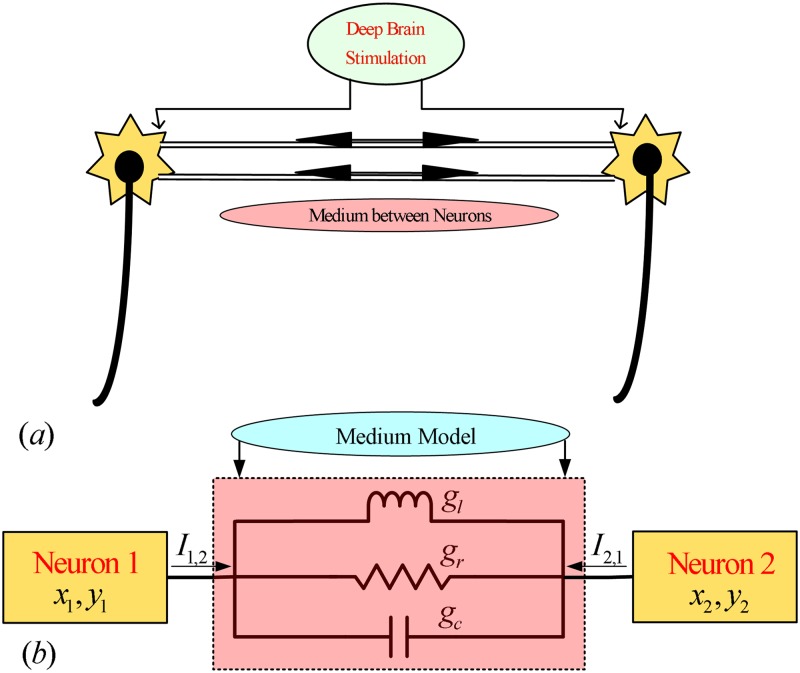
Modeling of medium between electrically coupled FHN neurons. The electrical signals between the neurons can pass through the inter-neuronal medium. This medium has resistive, inductive and capacitive characteristics. Therefore, it can be modeled through an RLC circuit: (a) Two neurons under external stimulation and communicating through the medium, (b) neuronal signals facing the RLC medium.

A neuronal coupling medium senses the electrical pulses (known as neuronal signals or spikes) generated by neurons during neuronal activities and transmits them to other neurons. The coupling medium properties are represented by gap junctions, and the strength of these gap junctions depends linearly on the difference between the membrane potentials [17, 32–33]. Given that the properties of the coupling medium can affect neuronal synchronization, the behavior of gap junctions is studied by utilizing a neuronal model such as the FHN system under external electrical stimulation (e.g., deep brain stimulation). The schematic diagram in Fig 1 illustrates the coupling medium modeling scenario by considering electrically coupled FHN neurons under deep brain stimulation. Fig 1(a) demonstrates communication between two FHN neurons through the neuronal coupling medium under deep brain stimulation. Fig 1(b) more precisely focuses on the coupled model that incorporates the resistive, capacitive and inductive properties of the medium. The conventional coupling medium models, as in [24], [25], [31], [32], and [33], consider only the resistive properties of the coupling medium. However, when information is shared between two neurons separated by a coupling medium containing gap junctions, the electrical pulses face both capacitive and inductive effects in addition to the resistive property of the coupling medium, as seen in [18–20]. The present study explores a more general model of the coupling medium than the conventional approaches [24–25], [31–33]. In addition, the effects of the additional coupling medium terms on the coupled neurons and compensation of the medium strength are also investigated.

The hypothesis of inclusion of the inductive term in coupling medium can be justified through the famous phenomenon of electromagnetic induction discovered by Michael Faradays [42], which reveals that a magnetic field varying with time induces a voltage, which causes a current flow in a closed circuit. It is considered as a basic principle of inductors.

There are two types of neuronal transmission through which neurons can communicate: chemical synapses and electrical synapses. Regarding chemical synapses that permit unidirectional communication between neurons, it is true that this type of synapses cannot be modeled through an inductor. However, in electrical synapses (another type of synaptic junctions, that is, gap junctions) that permit bidirectional communication between neurons, they can be modeled through inductors together with resistors and capacitors. In this type of synapses, the communication between one neuron to another is direct; without chemical intervention, owing to the adjacency of the membranes of pre-synaptic and post-synaptic neurons. A neuronal membrane separates the inside part of a cell from the extracellular medium and acts as a partially porous fence to the diffusion of ions. Normally, the interior of a neuronal cell contains electrical charges; positive or negative, however, the negative charges have excess concentration in the inside of a neuron than the outside region. This concentration difference of negative and positive charges between the intracellular and extracellular mediums results in the generation of an electrical potential difference, called membrane potential. In more detail, it is evident that the membrane potential is generated by four relevant types of ions: Na+, K+, Cl^-^, and organic ions (A). A large number of organic ions cannot diffuse through the membrane; however, some of the ions like Na+ and K+ can pass through intra membranous protein holes, called ionic channels, owing to the partial permeability of the membrane. The concentrations of K+ and Na+ are different inside and outside regions of the membrane. The K+ ions are more concentrated in the inside region, while the Na+ ions have more concentration in the extracellular medium. A chemical concentration gradient makes K+ ions to flow towards the extracellular medium, while a potential difference, produced owing to the separation of the electrical charges, tends to make K+ ions to flow back to the inside part of a neuron. A similar diffusion process can be observed for Na+ ions. The out flux of K+ ions to the extracellular medium from the inside region polarizes the membrane and the influx of Na+ to the intracellular medium depolarizes the membrane. In reality, the membrane responses to the potassium activation and sodium inactivation result in the flow of ionic current. The process of ionic current flow from one neuron to another is the same as per Faraday’s experimental results. These ions (potassium and sodium) carrying electrical charges produces a time-varying field, which develops an induced voltage to produce a flow of current. Faraday’s law is the fundamental principle of inductors, and these inductors are characterized by their inductances. Therefore, the physical phenomenon suggests the existence of an inductive property of the coupling medium.

A combination of the three effects of resistive, inductive, and capacitive components leads to a parallel RLC circuit attributable to the electrical properties of the coupling medium. It is important to mention that the consideration of a parallel RLC circuit as a theoretical justification is based on a simple hypothesis: The RLC circuit consists of three electronic components (resistor, capacitor, and inductor) that represent the characteristics of the extracellular medium. The electrical behavior of this extracellular medium is assumed to be linear for simplicity. Owing to the linearity property, the principle of superposition is applicable to the RLC circuit, which results in a parallel configuration of the electronic elements and the total current passing through one end to the other of a parallel RLC circuit is the sum of the three current components. This is why it is more appropriate to consider a parallel RLC circuit instead of other circuit configurations. Accordingly, Fig 1(b) presents an electrical model of the neuronal coupling medium that consists of a resistor, a capacitor, and an inductor, representing the well-known parallel RLC circuit, faced by the electrical pulses transmitted between neurons. The electrical behavior of the inter-neuronal coupling medium is not only resistive but also further a function of resistance, capacitance, and inductance.

### Modeling of coupling medium between neurons

For derivation of a mathematical model of the coupling medium, consider two different FHN neurons (see also [31–33]) describing the coupling phenomenon under certain coupling medium properties:

$$\begin{array}{l} \frac{dx_{1}}{dt}=x_{1}(x_{1}-1)(1-r_{1}x_{1})-y_{1}-I_{1,2}+(a/\omega )\text{cos}(\omega t),\\ \frac{dy_{1}}{dt}=b_{1}x_{1}, \end{array}$$ (1)

$$\begin{array}{l} \frac{dx_{2}}{dt}=x_{2}(x_{2}-1)(1-r_{2}x_{2})-y_{2}-I_{2,1}+(a/\omega )\text{cos}(\omega t),\\ \frac{dy_{2}}{dt}=b_{2}x_{2}, \end{array}$$ (2)

where *x*_1_ and *y*_1_ are the states of the first FHN neuron in terms of the activation potential and the recovery variable, respectively, and *x*_2_ and *y*_2_ are the corresponding states of the second FHN neuron. The FHN model parameters (*r*_1_, *r*_2_) and (*b*_1_, *b*_2_) are linked with the nonlinear part and the recovery variable of the neurons, respectively. The parameter *a* denotes the amplitude of the external stimulation current for the master and slave neurons, respectively. Time and the angular frequency of the stimulation current are represented by *t* and *ω* = 2*πf*, respectively, where *f* denotes the frequency. *I*_1,2_ and *I*_2,1_ are the currents from the first to the second and from the second to the first neurons, respectively.

Primarily, the conduction currents were taken to be *I*_1,2_ = g(*x*_1_ − *x*_2_)*gx*_1,2_ and *I*_2,1_ = g(*x*_2_ − *x*_1_)*gx*_2,1_, where *g* is the coupling medium’s resistive conduction strength, as seen in the literature ([24], [25], and [31–33]). In this paper, however, as per the proposed electrical structure of the coupling medium, *I*_1,2_ is taken to have the form

$$I_{1,2}=i_{R}+i_{C}+i_{L},$$ (3)

where *i*_*R*_, *i*_*C*_ and *i*_*L*_ represent the resistive, capacitive, and inductive currents given by

$$\begin{array}{l} i_{C}=g_{c}\frac{dx_{1,2}}{dt},\\ i_{R}=gx_{1,2},\\ i_{L}=g_{l}\int_{0}^{t}x_{1,2}dt. \end{array}$$ (4)

Here, *g*_*c*_ and *g*_*l*_ are the capacitive and inductive strengths of the coupling medium. By utilizing Eqs (3) and (4), the conduction current *I*_1,2_ becomes

$$I_{1,2}=gx_{1,2}+g_{c}\frac{dx_{1,2}}{dt}+g_{l}\int_{0}^{t}\left(x_{1,2}\right)dt.$$ (5)

And similarly, the expression for *I*_2,1_ takes the form

$$I_{2,1}=gx_{2,1}+g_{c}\frac{dx_{2,1}}{dt}+g_{l}\int_{0}^{t}x_{2,1}dt.$$ (6)

Eqs (5) and (6) represent the mathematical model of the inter-neuronal coupling medium in terms of the flow of current between neurons; thereby they generalize the conventional coupling medium model given by *I*_1,2_ = *gx*_1,2_ and *I*_2,1_ = *gx*_2,1_. By ignoring the capacitive and inductive effects, that is, *g*_*c*_ = 0 and *g*_*l*_ = 0, Eqs (5) and (6) are reduced to the traditional model given by *I*_1,2_ = *gx*_1,2_ and *I*_2,1_ = *gx*_2,1_.

By substituting Eqs (5) and (6) into Eqs (1) and (2), a generalized model of coupled FHN neurons can be obtained as

$$\begin{array}{l} \frac{dx_{1}}{dt}=x_{1}(x_{1}-1)(1-r_{1}x_{1})-y_{1}-g(x_{1}-x_{2})-g_{c}({\dot{x}}_{1}-{\dot{x}}_{2})\\ \;-g_{l}\int_{0}^{t}\left(x_{1}-x_{2}\right)dt+(a/\omega )\text{cos}(\omega t),\\ \frac{dy_{1}}{dt}=b_{1}x_{1}, \end{array}$$ (7)

$$\begin{array}{l} \frac{dx_{2}}{dt}=x_{2}(x_{2}-1)(1-r_{2}x_{2})-y_{2}-g(x_{2}-x_{1})-g_{c}({\dot{x}}_{2}-{\dot{x}}_{1})\\ \;-g_{l}\int_{0}^{t}\left(x_{2}-x_{1}\right)dt+(a/\omega )\text{cos}(\omega t),\\ \frac{dy_{2}}{dt}=b_{2}x_{2}. \end{array}$$ (8)

In contrast to the conventional coupled neuronal systems ([24], [25], [31–33]), models (7) and (8) represents a more practical form of coupled neurons, by incorporating the coupling medium properties and showing that a parallel RLC circuit can exist between the neurons and such circuit would be responsible for neuronal communication. Primarily, the gap junctions permit communication between two neurons by capturing the coupling medium’s property of resistivity. However, the behavior of gap junctions and the coupling medium can be capacitive and inductive as well. To grasp these characteristics of the coupling medium, the medium of the modified FHN models (7) and (8) is characterized by the parameters *g*, *g*_*c*_, and *g*_*l*_. The consideration of the coupling medium as capacitive and inductive, in contrast to the literature ([12–17], [32–37]), provides a more realistic coupled FHN system and, hence, a more natural coupled neuronal model.

### Coupling strength as PID control for synchronization

After obtainment of a model of the underlying coupling medium between neurons, the model is used to investigate the natural phenomenon of the synchronization of potentials in a neuronal network. The mathematical equations for the coupling medium reveal the existence of natural three control terms known as the proportional integral derivative (PID) controller. To demonstrate the existence of a natural PID control between neurons, we assign

$$f_{1}(x_{1})=x_{1}(x_{1}-1)(1-r_{1}x_{1}),$$ (9)

$$f_{2}(x_{2})=x_{2}(x_{2}-1)(1-r_{2}x_{2}),$$ (10)

$$u_{pid}(e)=k_{p}e+k_{i}\int_{0}^{t}edt+k_{d}\dot{e},$$ (11)

where *e* = *x*_1,2_ represents the error between activation potentials of neurons, and *k*_*p*_ = *g*, *k*_*i*_ = *g*_*l*_, and *k*_*d*_ = *g*_*c*_ refer to the proportional, integral, and derivative gains due to the resistive, inductive, and capacitive properties of the coupling medium, respectively. The signal *u*_*pid*_(*e*) = *I*_1,2_ denotes the current due to the coupling between neurons in terms of a PID control signal obtained by combining the proportional, integral, and derivative operations on the synchronization error. Employing Eqs (9)–(11), we re-write the coupled FHN neurons in Eqs (7) and (8) as

$$\begin{array}{l} \frac{dx_{1}}{dt}=f_{1}(x_{1})-y_{1}+(a/\omega )\text{cos}(\omega t)-u_{pid}(e),\\ \frac{dy_{1}}{dt}=b_{1}x_{1}, \end{array}$$ (12)

$$\begin{array}{l} \frac{dx_{2}}{dt}=f_{2}(x_{2})-y_{2}+(a/\omega )\text{cos}(\omega t)+u_{pid}(e),\\ \frac{dy_{2}}{dt}=b_{2}x_{2}. \end{array}$$ (13)

The models (12) and (13) reveals that the neurons receive a feedback of the error signal *e* = *x*_1_ − *x*_2_, which is processed by the resistive, inductive, and capacitive effects of the coupling medium as *u*_*pid*_(*e*) and forms a PID controller. It is notable that the dynamics of FHN neurons Eqs (12) and (13) containing $u_{pid}(e)=k_{p}e+k_{i}\int_{0}^{t}edt+k_{d}\dot{e}$ confirms the existence of a PID controller in the model of coupled neurons.

**Fig 2:**
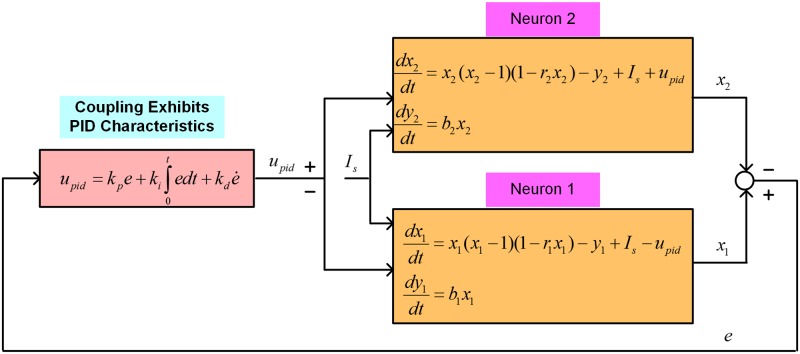
The existence of a natural PID control between FHN neurons coupled through a medium. This medium, forming an RLC circuit, can be represented as natural resistive, inductive, and capacitive properties, which are responsible for the regulation of the synchronous behavior of the neurons. The natural PID control can be understood as a mechanism for tracking the behavior of one neuron via another.

The schematic diagram in Fig 2 shows the existence of a natural PID controller in the model of coupled FHN neurons when assigning *I*_*s*_ = (*a*/*ω*)cos(*ωt*). The occurrence of feedback and PID control can easily be verified for Neuron 2, as the error signal *e* is employed as a feedback through the PID controller formed by the coupling terms. Similarly, the existence of a natural PID controller due to the coupling medium properties can be verified for Neuron 1. The functioning of PID control in neurons can ensure the tracking of the activation potential of Neuron 1 by that of Neuron 2, and vice versa. The synchronization phenomenon between the neurons can be explained in terms of the tracking of the activation potential of a neuron by another neuron through a PID controller. Hence, a PID controller exists between two neurons for their synchronization, and the entirety of information processing in the brain is controlled through multiple PID controllers formed due to communication between neurons via the coupling medium and its resistive, inductive, and capacitive properties.

### Effects of PID components in neuronal synchronization

The PID phenomenon existing between neurons acts as a feedback control device that can enhance the synchronization process in the brain. The numerical simulation showed that the steady-state performance is within an acceptable limit and that the transient response follows the variations in the PID components. The model parameters are selected as *r*_1_ = 10, *r*_2_ = 10.5, *b*_1_ = 1, *b*_2_ = 1.2, *ω* = 0.8796, and the stimulation amplitude *α* = 0.1 for different values of *g*, *g*_*c*_ and *g*_*l*_ in terms of *k*_*p*_, *k*_*i*_ and *k*_*d*_. Please refer to the next section for the simulation method and software details, which demonstrates the effectiveness of the PID components on synchronization.

**Fig 3:**
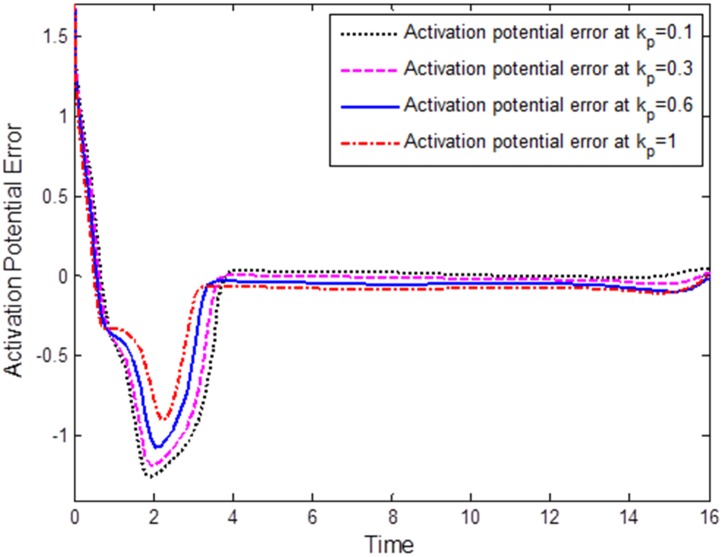
Comparison of the errors under various proportional components on the synchronization of FHN neurons: *k*_*p*_ = 0.1, *k*_*p*_ = 0.3, *k*_*p*_ = 0.6, *k*_*p*_ = 1. The synchronization error decreases with increasing *k*_*p*_; however, an increase in the overshoot is seen as *k*_*p*_ gets smaller.

The first numerical simulation was conducted to verify the effect of the proportional component *k*_*p*_ of the PID on synchronization of FHN neurons. For this, we fixed the integral and derivative constants of the natural PID as *k*_*i*_ = 0.0001 and *k*_*d*_ = 0.00001. The effects of *k*_*p*_ on synchronization of FHN neurons can be observed in Fig 3, which shows the synchronization error plots for different values of the proportional component *k*_*p*_. For a small value of *k*_*p*_ = 0.1, the FHN neurons incur a large synchronization error and show a slow convergence. As we increase the value of the proportional component to *k*_*p*_ = 0.3, the synchronization error decreases in the steady state and approaches to zero. When *k*_*p*_ = 0.6, the neurons exhibited almost synchronous behavior. However, for *k*_*p*_ = 1, the steady-state synchronization error further decreased. It is evident from Fig 3 that the increased proportional gain *k*_*p*_ of the natural PID decreases the steady-state synchronization error between the FHN neurons with a large overshoot.

**Fig 4:**
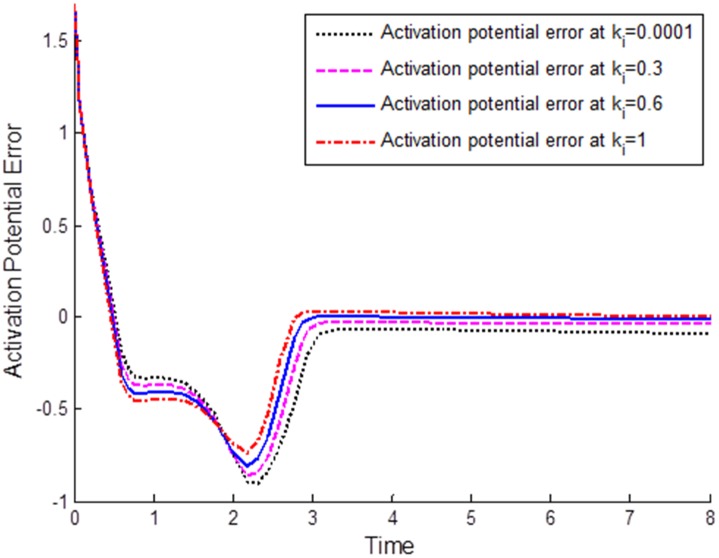
Comparison of integral components to synchronization error: *k*_*i*_ = 0.0001, *k*_*i*_ = 0.3, *k*_*i*_ = 0.6, *k*_*i*_ = 1. By increasing the value of *k*_*i*_, the synchronization error in the steady state can be reduced to zero. That is, a complete synchronization between neurons can be attained for a large value of the integral constant of the natural PID control.

In the second numerical simulation, we studied the effects of integral component *k*_*i*_ of the natural PID controller on the synchronization of the coupled FHN neurons. The other parameters chosen were *k*_*p*_ = 1 and *k*_*d*_ = 0.00001. Fig 4 plots the synchronization errors under various values of integral component *k*_*i*_. At the very small value of *k*_*i*_ = 0.0001, both FHN neurons showed a certain synchronization error; however, as the value increased to *k*_*i*_ = 0.3, the error decreased, and by increasing *k*_*i*_ further to *k*_*i*_ = 0.6 and after once more time to *k*_*i*_ = 1, the steady-state error became zero. It is observed that the synchronization error approaches to zero as integral gain *k*_*i*_ increases. Hence, the integral component of the natural PID control can eliminate the synchronization error between neurons, and thus plays a vital role in attainment of neuronal synchronization.

**Fig 5:**
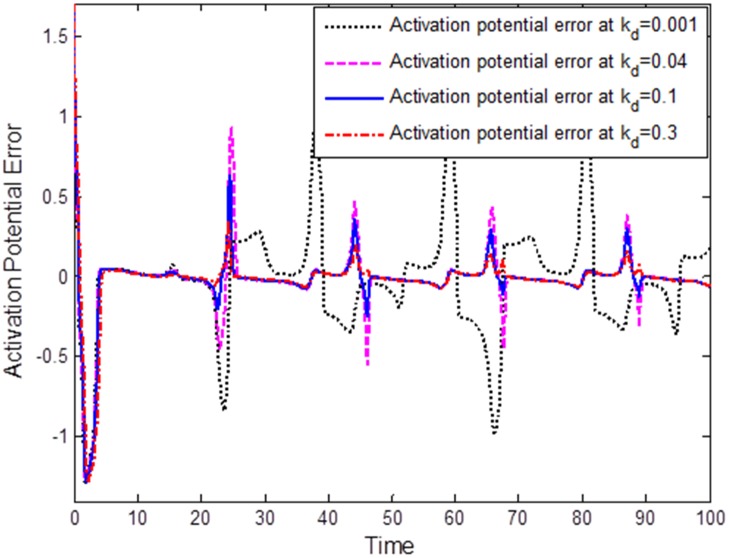
Comparison of derivative components to synchronization error: *k*_*d*_ = 0.001, *k*_*d*_ = 0.04, *k*_*d*_ = 0.1, *k*_*d*_ = 0.3. By increasing the value of *k*_*d*_, the oscillatory effects of the synchronization error can be reduced. The derivative component of the natural PID control can improve the stability of the synchronization error system.

In the third numerical simulation, we investigated the effects of the derivative component *k*_*d*_ of the PID on synchronization of FHN neurons. The proportional and integral constants were taken as *k*_*p*_ = 0.01 and *k*_*i*_ = 0.01, respectively. Fig 5 plots the synchronization error under different parametric values of *k*_*d*_ in the natural PID control. Initially, we chose *k*_*d*_ = 0.001; at this small value, the synchronization error oscillated, leading to a non-synchronous neuronal behavior. However, at *k*_*d*_ = 0.04, *k*_*d*_ = 0.1 and, further, up to *k*_*d*_ = 0.3, the overall synchronization error response was stable, with almost zero steady-state synchronization error. This indicates that the derivative component of the synchronization error system can improve system stability.

The following important conclusion can be drawn from the simulation results, Figs 3–5: The standard characteristics of a PID control, namely zero steady-state error, fast response, and good stability properties, can easily be verified in terms of the synchronization error for FHN neurons communicating through a coupling medium having resistive, capacitive, and inductive properties. The components of the natural PID controller are responsible for regulation of the synchronization error in the membrane potentials.

### Compensation of natural coupling strength deficiency for synchronization

To cover the strength deficiency of the PID components, an additional control signal from another PID controller can be incorporated. This new control signal is added to the second neuron, and accordingly, the dynamics of the overall FHN systems are given as

$$\begin{array}{l} \frac{dx_{1}}{dt}=f_{1}(x_{1})-y_{1}+(a/\omega )\text{cos}(\omega t)-u_{pid},\\ \frac{dy_{1}}{dt}=b_{1}x_{1}, \end{array}$$ (14)

$$\begin{array}{l} \frac{dx_{2}}{dt}=f_{2}(x_{2})-y_{2}+(a/\omega )\text{cos}(\omega t)+u_{pid}+u_{s},\\ \frac{dy_{2}}{dt}=b_{2}x_{2}. \end{array}$$ (15)

The supervisory control signal *u*_*s*_ should be designed in such a way that the synchronization error, owing to the natural coupling strength deficiency, convergences either to zero or to within a small compact set around zero. We hypothesized that the signal *u*_*s*_ can be delivered to a neuron through some feedback mechanism of electrical stimulation or drug delivery in a similar manner as in the literature ([24], [25], [31–33]). In order to enhance the synchronization procedure in nervous systems, this paper proposes a supervisory mechanism for compensation of the natural coupling strength deficiency of FHN neurons. The control signal *u*_*s*_ generated from the supervisory mechanism can effectively synchronize the master-slave FHN neurons Eqs (3) and (4) via a capacitive and inductive medium. Using *e* = *x*_1_ − *x*_2_, the dynamics in Eqs (14) and (15) reveals

$$\frac{de}{dt}=f_{1}(x_{1})-f_{2}(x_{2})-y_{1}+y_{2}-(2u_{pid}+u_{s}).$$ (16)

Hence, the new signal *u*_*s*_, owing to the term 2*u*_*pid*_ + *u*_*s*_ in the synchronization error dynamics, improves the strength of the existing natural PID signal *u*_*pid*_. Here, the selection of *u*_*s*_ becomes critical for attainment of synchronization between the neurons. In principle, the supervisory mechanism must be selected as

$$u_{s}={\tilde{k}}_{p}e+{\tilde{k}}_{i}\int_{0}^{t}edt+{\tilde{k}}_{d}\dot{e},$$ (17)

which improves the strength of the natural PID components *k*_*p*_, *k*_*i*_ and *k*_*d*_ through ${\tilde{k}}_{p},$ ${\tilde{k}}_{i}$ and ${\tilde{k}}_{d}$, respectively.

### PSO-based supervisory mechanism

Incorporation of a PID controller to address the coupling strength deficiency for synchronization of neurons is interesting due to its ability in improving the natural PID components; however, obtainment of the supervisory PID controller components is a challenging task. In neuronal systems, almost all the neural parameters are unknown, and even stimulation parameters are uncertain due to medium losses and unavoidable phase shifts. The coupling medium properties also vary with time, resulting in fluctuations in the natural PID parameters *k*_*p*_, *k*_*i*_ and *k*_*d*_. Supervisory mechanism Eq (17) for neuronal synchronization must be designed to cope with such uncertainties and variations. For this purpose, we employed the particle swarm optimization (PSO) technique to find the supervisory mechanism parameters ${\tilde{k}}_{p},$ ${\tilde{k}}_{i}$ and ${\tilde{k}}_{d}$. The beauty of the PSO algorithm is that it does not require the values of the neuronal, stimulation, and coupling medium parameters for adaptive determination of the parameters to attain optimization of a desired objective function.

**Fig 6:**
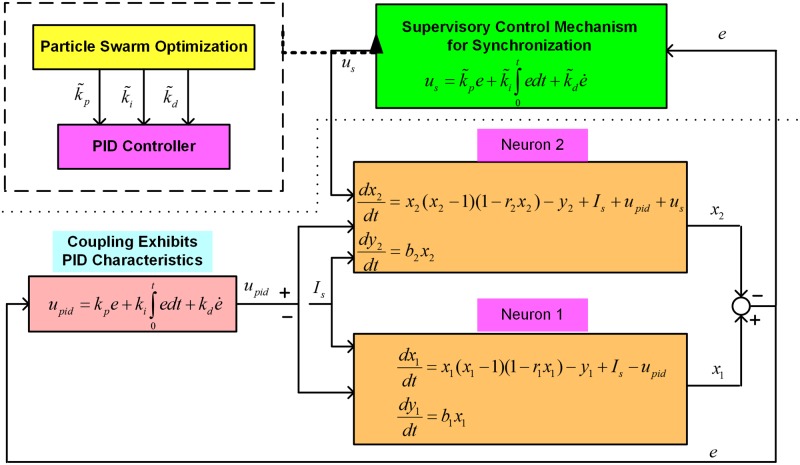
Supervisory mechanism for PID-controlled synchronization of neurons. By employing PSO and an additional PID control strategy, the coupling deficiency between the neurons can be improved. The proposed controller can increase the strength of the natural PID mechanism. The parameters of the new PID control can be tuned through adaptation for attainment of synchronization. PSO is employed to determine the optimal PID parameters for neuronal synchronization.

The overall closed-loop system formed by the FHN neurons Eqs (14) and (15), coupling medium Eq (11), and supervisory PID mechanism Eq (17) is shown in Fig 6. The two systems of the FHN neurons are in coordination through a coupling PID according to the intrinsic properties of the coupling medium, while an additional feedback signal *u*_*s*_ is added to the neurons through a supervisory mechanism. The neuronal coupling medium exhibiting natural PID characteristics can be characterized by parameters *k*_*p*_, *k*_*i*_ and *k*_*d*_. The neurons communicating through the coupling medium might exhibit an asynchronous behavior owing to the natural coupling strength deficiency. A supervisory mechanism augmented in the system of FHN neurons can compensate for the natural coupling strength deficiency and as such represent an effectual means of improving coordination in neuronal functions.

**Fig 7:**
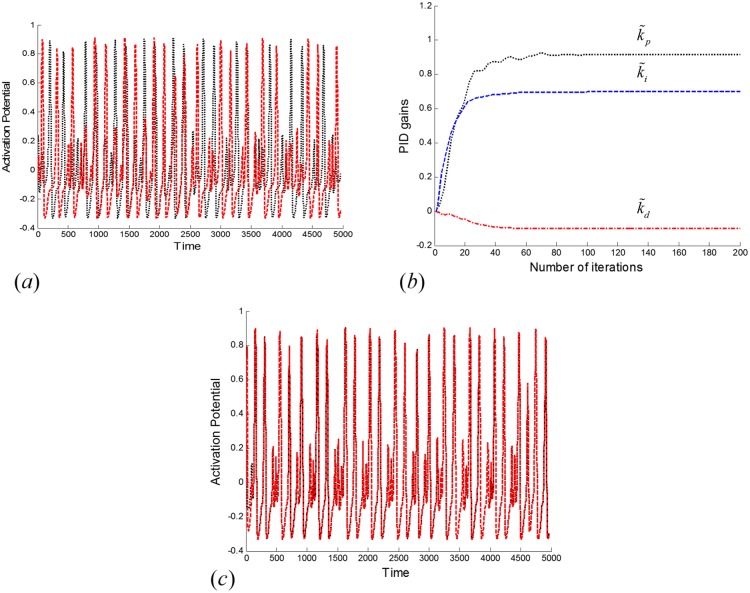
Controlled synchronization by application of the proposed supervisory mechanism. By application of this mechanism, non-synchronous neurons can be adaptively synchronized through adaptation of the proportional, integral, and derivative components: (a) Non-coherent activation potentials without the supervisory mechanism, (b) PID gains’ convergence curves for the proposed PSO approach, and (c) activation potentials using the optimal gains ${\tilde{k}}_{p}=0.9$, ${\tilde{k}}_{i}=0.7$ and ${\tilde{k}}_{d}=-0.1$ of the proposed supervisory mechanism.

The supervisory mechanism, which works according to the policy of learning and adaptation, solves the parametric estimation problem of the supervisory PID controller by utilizing the PSO technique to ensure synchronization. The optimum parameters ${\tilde{k}}_{p}$, ${\tilde{k}}_{i}$ and ${\tilde{k}}_{d}$ of the PID controller are obtained for the convergent minimum value of the objective function defined as

$$J=\int_{0}^{\tau }e^{2}dt,$$ (18)

where *τ* is the time of integration and *J* is the objective function. Let *α* ∈ *S* be defined such that $\alpha =\left[\begin{array}{ccc} {\tilde{k}}_{p} & {\tilde{k}}_{i} & {\tilde{k}}_{d} \end{array}\right]$ represents a particle, where *s* = {*α* ∈ *R*^3^; 0 ≤ *α*_*i*_ ≤ *α*_max_, *i* = 1,…, 3} is the search area. The optimum solution for the parametric vector *α* is such that α* ∈ *S* minimizes the objective function *J*. The simulation results in the subsequent plots of Fig 7 verify the efficacy of the proposed supervisory mechanism. The three derived optimal gains were ${\tilde{k}}_{p}=0.9$, ${\tilde{k}}_{i}=0.7$ and ${\tilde{k}}_{d}=-0.1$ to shape the output signal *u*_*s*_ of the supervisory control mechanism for synchronization of the FHN neurons by compensation of the natural coupling strength deficiency. Fig 7(a) plots the activation potentials of both neurons (dashed line for *x*_1_, dotted line for *x*_2_) without any supervisory mechanism. Both activation potentials show non-coherent behaviors due to strength deficiency in natural coupling between the neurons. The adaptive estimation of the supervisory mechanism gains ${\tilde{k}}_{p}$, ${\tilde{k}}_{i}$ and ${\tilde{k}}_{d}$ for compensation of the natural coupling strength deficiency is indicated in Fig 7(b). By the PSO technique, these parameters converged to ${\tilde{k}}_{p}=0.9$, ${\tilde{k}}_{i}=0.7$ and ${\tilde{k}}_{d}=-0.1$. By application of the proposed supervisory mechanism, the activation potentials of both FHN neurons were synchronized as shown in Fig 7(c). Hence, it can be concluded that the proposed PSO-based approach is effective for neurons’ synchronization via estimation of the coupling strength deficiency between them.

The aim of this paper is to address the modeling of the coupling medium and its behavior at a very basic level of two neurons to provide a more fundamental model. In the future, the proposed methods can be extended to the synchronization of multiple neurons with complex coupling paradigm.

## Methods

### Faraday’s law of induction

Discovery has played a vital role in the development of the modern electrical technologies. Faraday’s law is a fundamental theory that acts as a working principle for many of the devices such as inductor, electric motors, and generators and provides a foundation for the evolution of applied science and real-world engineering. Faraday’s law states that a time-varying magnetic field establishes an induced voltage in a closed circuit, which causes a flow of induced current [42].

### Principle of superposition

The principle of superposition has a variety of applications in many fields such as engineering and physics. The method provides an easier way to analyze and understand complex systems. It is based on the homogeneity and additive properties. Mostly, it is applicable to the physical systems that can be modeled as linear systems. The significance of a linear system is that it can be analyzed easily. By definition, the superposition principle simply states that, for a linear system, the overall effect of several loads applying at the same time is equal to the algebraic sum of the effects of individual loads acting simultaneously [43].

### FitzHugh-Nagumo model

Neurons are the principal functional units of the brain; as such, their dynamical investigation is central to the investigation and treatment of various brain disorders [25–27]. Accordingly, a variety of mathematical models have been developed to capture neurons’ firing behaviors in the brain. Hodgkin-Huxley, Hindmarsh-Rose, and FitzHugh-Nagumo are the most successful models in the dynamical systems perspective of neurons in terms of chaos, bifurcation, oscillations, spikes, and other complex behaviors. The FitzHugh-Nagumo (FHN) model, under sinusoidal electrical stimulation, is widely utilized in neuronal synchronization studies due to its utility in representing the different dynamical aspects of neurons. Consider the following FHN model of a neuron under external electrical stimulation [31]:

$$\begin{array}{l} \frac{dx}{dt}=x(x-1)(1-rx)-y+(a/\omega )\text{cos}\omega t,\\ \frac{dy}{dt}=bx+vy, \end{array}$$ (19)

where *x* and *y* are the states of the FHN neuron in terms of activation potential and recovery voltage, respectively. Parameter *r* represents the nonlinearity in the model, *b* and *v* are the parameters linked with the recovery voltage, and parameter *a* indicates the amplitude of the external stimulation current. We utilized this fundamental neuronal model to study the effects of the coupling medium on neuronal synchronization.

### Synchronization

By definition, synchronization is the adjustment of the rhythms of oscillating systems through their (weak) interactions [44]. There are three requirements for synchronization: (i) periodic or aperiodic oscillatory motion of systems, (ii) unidirectional or bidirectional interaction between them, and (iii) the rhythms of the oscillatory systems must be adjusted. In brain dynamics, neuronal firing is independent and simultaneous; however, synchronization eventually occurs [45]. Usually, synchronization is observed in different areas of the brain such as the visual cortex and the olfactory bulb (see [46–48]). This section demonstrates some fundamental mathematical essentials of synchronization. Consider the nonlinear system

$$\begin{array}{l} \frac{dx}{dt}=f(t,x),\\ x(0)=x_{0}. \end{array}$$ (20)

Now, consider another nonlinear feedback control system, given by

$$\begin{array}{l} \frac{dy}{dt}=g(t,y)+bu,\\ y(0)=y_{0}, \end{array}$$ (21)

where $x\in {ℜ}^{n}$ and $y\in {ℜ}^{n}$ represent the states of the systems. The nonlinear functions $f(t,x)\in {ℜ}^{n}$ and $g(t,y)\in {ℜ}^{n}$ are time-varying vectors. Here, $u\in {ℜ}^{m}$ represents the control input vector. The initial conditions are taken as *x*_0_ and *y*_0_. The above-mentioned systems in Eqs (20) and (21) are said to be synchronized, if the synchronization error *e* = *x* − *y* converges to zero. The synchronous behavior of the systems can be achieved through a proper selection of control input *u*.

### Proportional integral derivative (PID) control

Proportional integral derivative (PID) control is a well-known technique in various domains of engineering [38–41] for regulation of the behavior of a dynamical system. It can be expressed as

$$u_{pid}=k_{p}e+k_{i}\int_{0}^{t}edt+k_{d}\dot{e},$$ (22)

where *k*_*p*_, *k*_*i*_ and *k*_*d*_ are the PID controller’s proportional, integral, and derivative parameters, respectively. The synchronization error signal *e* is shaped by individual PID terms *k*_*p*_, *k*_*i*_ and *k*_*d*_. For instance, error *e* is proportional to the controller in that as it increases, the feedback controller devotes more effort to reduce it. The integral term has to guarantee the zero steady-state error, but it can cause often an overshoot. The derivative term, contrastingly, has marvelous stability properties; therefore, it is required to eliminate the oscillations.

### Simulation method and software

In this modern era of technology, the state-of-the art methods in modeling and simulation of engineering systems have received more attention owing to their extensive support to the design and development process. Modeling and simulation help to achieve the desired goal virtually rather than through physical experiments. Simulation experimental studies have a variety of applications in multi-disciplinary systems in which different components are tightly coupled to attain an optimal performance. In neuroscience studies (or computational neuroscience), modeling and simulation is more useful owing to the more physical limitations (difficulties in physical experimental measurements). Indeed, simulation experimentation has emerged as a key tool of dynamical analysis in contemporary research, especially in the field of neuroscience. In the present work, numerical simulations were performed with a mathematical model to explore the neuronal coupling medium properties to understand the impact of the coupling medium on the synchronization of the neuronal potentials in a neuronal network.

For simulation of coupled FHN models, a complete SIMULINK/MATLAB model was developed. Functions can be written in any programming language for SIMULINK model. However, for a built-in MATLAB S-function, any familiar function of MATLAB can also be used. S-functions, written in programming languages like C, are employed to embed an object code into a SIMULINK model. There are two steps: First, the FHN models are programmed using S-functions with easily adjustable coupling parameters of model. In the second step, the S-functions are called into the SIMULINK model. Using this approach, numerical simulation on the effects of the coupling medium on synchronization was performed.

### Particle swarm optimization (PSO)

Particle swarm optimization (PSO) is well known for its provision of an alternative solution to nonlinear complex optimization problems. It has gained an interest from the researchers for its simplicity of implementation as well as its ability to swiftly converge to an optimal solution. Inspired by the social behavior of bird flocks, it finds the optimal solution by exploiting the population of particles in a search space [49], see also [38–41]. In this method, each particle moves in the search domain and updates its velocity, according to both its own flying experience and that of its neighbors, toward its personally and globally best location. Rather than the conventional methods of [46–48] and [50–51], we have employed PSO in our analysis for the synchronization of neurons. The fundamental PSO algorithm is as follows:

1. Generate a population of particles and initialize their positions and velocities randomly.
2. Evaluate the objective function for each particle and compare each fitness value with the particle’s personal best (pbest). If the current fitness is better than the pbest, assign the pbest value to the current one and the pbest location to the current location.
3. Likewise record the global best position (gbest).
4. Update each particle’s velocity and position according to the given equations
  $$V_{i}=WV_{i}+C_{1}R_{and}(P_{best}-X_{i})+C_{2}R_{and}(G_{best}-X_{i}),$$ (23)
  $$X_{i}=X_{i}+V_{i},$$ (24)
  which define the PSO algorithm’s key mechanism. Here, *V*_*i*_ and *X*_*i*_ are the velocity and position of the *i*th particle, and *W* is the inertial weight, which balances the searches. The learning constants are denoted by *C*_1_ and *C*_2_. *P*_*best*_ is the current optimal position of each particle, and *G*_*best*_ is the current optimal position among all particles.
5. Return to step (ii) until the stop criterion (i.e., a good fitness) is achieved.

## Conclusions

In this paper, we discussed a new inter-neuronal coupling medium model and introduced a controlling mechanism based on the policy of learning and adaptation to ensure the synchronization of various processes in the brain. This approach is of interest for several reasons. First of all, a neuronal coupling medium model that accords with the neuron’s intrinsic electrical properties was derived by utilizing the knowledge of electrical circuits and biological-experimental evidence. Amazingly, the incorporation of such properties revealed the existence of a natural three-term control strategy known as the proportional integral derivative (PID) controller. Further, we studied the impact of such properties on synchronization, and observed that brain-information processing can rely on a large number of PID controllers resulting from the coupling medium properties. The end product of the present research is the proposed novel scheme that compensates for the natural coupling strength deficiency by introducing a supervisory mechanism that works on the basis of a learning and adaptation policy and solves the parametric selection problem of the PID controller by utilizing the particle swarm optimization technique.
